# Enhanced Precision Time Synchronization for Wireless Sensor Networks

**DOI:** 10.3390/s110807625

**Published:** 2011-08-02

**Authors:** Hyuntae Cho, Jongdeok Kim, Yunju Baek

**Affiliations:** 1 Institute of Logistics Information Technology, Pusan National University, Geumjeong-gu, Busan 609-735, Korea; E-Mail: marine@pnu.edu; 2 Department of Computer Engineering, Pusan National University, Geumjeong-gu, Busan 609-735, Korea; E-Mail: kimjd@pnu.edu

**Keywords:** time synchronization, wireless sensor network, lightweight, hardware assisted time stamp, drift correction

## Abstract

Time synchronization in wireless sensor networks (WSNs) is a fundamental issue for the coordination of distributed entities and events. Nondeterministic latency, which may decrease the accuracy and precision of time synchronization can occur at any point in the network layers. Specially, random back-off by channel contention leads to a large uncertainty. In order to reduce the large nondeterministic uncertainty from channel contention, we propose an enhanced precision time synchronization protocol in this paper. The proposed method reduces the traffic needed for the synchronization procedure by selectively forwarding the packet. Furthermore, the time difference between sensor nodes increases as time advances because of the use of a clock source with a cheap crystal oscillator. In addition, we provide a means to maintain accurate time by adopting hardware-assisted time stamp and drift correction. Experiments are conducted to evaluate the performance of the proposed method, for which sensor nodes are designed and implemented. According to the evaluation results, the performance of the proposed method is better than that of a traditional time synchronization protocol.

## 1. Introduction

Time values provide the criterion to order events, their causal relationships and correlations, and the rate of change of the entities involved [1–3]. The provision and synchronization of times in distributed computing elements are common requirements for numerous distributed applications. WSNs consist of a number of sensor nodes, which are randomly deployed in the field. Time synchronization in WSNs is essential to efficiently monitor or gather sensing data and control sensor nodes [4–6]. Numerous time synchronization protocols for WSNs, LR-WPANs and *ad hoc* networks have been developed, e.g., RBS [7], TPSN [8], FTSP [9], *etc.* [10–19]. Traditional time synchronization protocols have struggled to provide precision time synchronization. Their efforts have largely focused on removing jitter at the protocol stack, correcting clock drift and considering a poor resource node. The channel contention needed for communication or time synchronization also affects the precision and accuracy of time synchronization. The random back-off delay for channel access can be a crucial uncertainty that is a nondeterministic error factor for the time synchronization procedure [12,13]. This error factor caused by the random back-off delay has to be considered for precise time synchronization.

First, we analyze the uncertainty that occurs at the network protocol stack during the synchronization procedure and then propose a novel approach based on the analyzed factors to reduce the nondeterministic latency. The main contribution of the proposed method is the reduction of the nondeterministic uncertainty by channel contention. To reduce this nondeterministic uncertainty, the proposed method reduces the traffic needed for the synchronization procedure by making sensor nodes selectively forward the messages needed for synchronization. The time difference between clocks increases as time advances because of the use of a clock source with a cheap crystal oscillator, even though the sensor nodes’ clocks are set to exactly the same frequency via synchronization. In addition, we provide a means to maintain accurate time by adopting hardware-assisted time stamp and drift correction. Furthermore, we evaluate the performance of the proposed method. We simulated the proposed method to examine the effect of the nondeterministic latency caused by channel contention. The proposed method not only dramatically reduced the number of messages needed for synchronization but also attained a higher precision than the traditional protocol. We also designed and implemented a sink node and sensor nodes to determine the performance increase by the precision time stamping technique and drift correction. An evaluation of the implemented nodes showed that the proposed method had 5-times better performance than a traditional time synchronization protocol in terms of precision.

The remainder of this paper is organized as follows. In Section 2, we classify time synchronization protocols into three types and present the traditional time synchronization protocols. In Section 3, we describe the proposed time synchronization protocol for distributed sensor nodes for WSNs. In Section 4, we present the system implementation and performance evaluation of the proposed method. In Section 5, we conclude the paper with plans for future work.

## 2. Related Work

In WSNs, the sensor nodes are usually scattered in a sensor field. Each of these scattered sensor nodes has the capability of collecting and routing data back to the sink and end users. Data is routed back to the end user using a multi-hop infrastructure-less architecture through the sink node. The sink may communicate with the remote user via the Internet or a satellite [20]. Sensor nodes synchronize their time with a reference clock such as the sink node or coordinated universal time (UTC), which is the time standard by which the world regulates clocks and time in time synchronization. Time synchronization in WSNs refers to the problem of synchronizing clocks across a set of sensor nodes that are connected to one another over single-hop or multi-hop wireless networks. Up to now, various protocols have been designed to address this problem [7–19].

**Figure 1.**
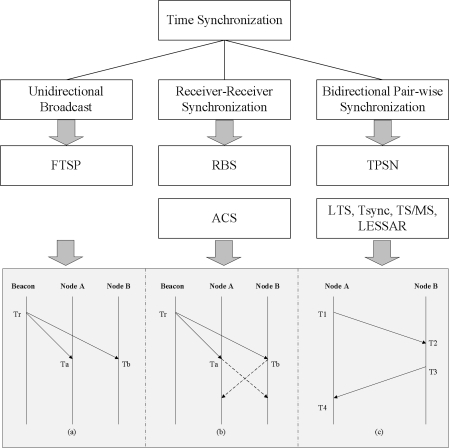
Classification of time synchronization protocols.

Time synchronization may be classified into three types: (a) simple unidirectional broadcast, (b) receiver-receiver synchronization and (c) bidirectional pair-wise synchronization, as shown in Figure 1. First, in the unidirectional reference broadcast method, a reference node simply broadcasts a reference clock signal to other nodes, which correct their times to match the reference clock. This method is the oldest and simplest method for synchronizing the network’s time. The flooding time synchronization protocol (FTSP) [9] is the most well-known approach. FTSP uses a fine-grained clock, MAC layer time stamping to reduce jitter and clock drift estimation to achieve relatively high precision.

Second, receiver-receiver synchronization uses an external beacon node. This beacon node periodically sends beacon messages to the sensor nodes. The sensor nodes that receive the beacon messages exchange the arrival times of the messages between themselves to compare and correct their clock. Reference broadcast synchronization (RBS) [7] and adaptive clock synchronization (ACS) [10] are based on the receiver-receiver synchronization protocol. RBS does not utilize an explicit timestamp; instead, receivers use the arrival times as points of reference for comparing their clocks, as shown in Figure 1(b). This approach directly removes two of the largest sources of non-determinism involved in message transmission: the transmission time and access time in the network protocol stack. ACS, which extends RBS, focuses on reducing the number of the messages used to exchange the message arrival times. In order to reduce the number of messages, the beacon node is used instead of the sensor node to gather and compare the message arrival times.

Third, bidirectional pair-wise synchronization, which can also be called sender-receiver synchronization, uses the round trip time of the message to correct the offset and propagation delay. This approach is performed using a handshake protocol between a pair of nodes. That is, in bidirectional pair-wise synchronization, sensor nodes achieve clock synchronization with their parent node, while receiver-receiver synchronization makes sensor nodes synchronize their clocks with other sensor nodes on the same level. In Figure 1(c) depicts an example of the basic operation, which includes three sequential phases. First, node A sends its local time at time *T1*, and node B receives the message at time *T2* and records its local time. Then, time *T2* is calculated as *T2 = T1 + d + δ*, where *δ* denotes a clock offset between two nodes and *d* is the propagation delay between them. Next, node B responds with an ACK message to node A with times *T2* and *T3*. After receiving the ACK message at time *T4*, node A determines time *T4* as *T4 = T3 + d* − *δ*. Finally, node A can calculate the clock offset and propagation delay between two nodes, as below:

(1) $$\begin{array}{l} d=\frac{\left[(T2-T1)+(T4-T3)\right]}{2}\\ \delta =\frac{\left[(T2-T1)+(T4-T3)\right]}{2} \end{array}$$

Timing-sync protocol for sensor networks (TPSN) [8], lightweight time synchronization (Tsync) [12], tiny-sync and mini-sync (TS/MS) [14] and level synchronization by sender, adjuster and receiver (LESSAR) [13] are famous bidirectional pair-wise synchronization protocols for WSNs, while the network time protocol (NTP) [19] is a wide-spread bidirectional pair-wise synchronization protocol used by the Internet. TPSN provides synchronization for an entire network. First, a node is elected as a synchronization master, and a spanning tree with the master at the root is constructed by flooding the network. In a second phase, nodes synchronize to their parent in the tree by means of round-trip synchronization. TSync has a centralized version, and a decentralized version. Both protocols exploit a dedicated radio channel for synchronization messages to avoid inaccuracies by packet collisions. LESSAR is able to achieve the accuracy limitation while retaining the characteristics of low power consumption, affordable storage and small computation complexity by reducing the packet transmissions. TS/MS uses multiple pair-wise round-trip measurements and a line-fitting technique to obtain the offset and drift of the two nodes, rather than directly calculating the offset.

Bidirectional pair-wise synchronization has the advantage that uncertainties at the network protocol stack and the propagation delay can be mitigated by the exchange messages. However, this approach requires additional traffic, and the number of messages increases with the network scale. That is, sensor nodes contend among themselves to access the channel. Thus, a busy channel leads to nondeterministic latency in the MAC layer and finally diminishes the accuracy and precision of time synchronization. In other words, the MAC checks whether the channel is clear before it sends the *sync* or ACK message. If the channel is busy, because of the number of messages, MAC waits for a random back-off period. After waiting for this random back-off period, the node resends the message, including the time stamp value. This delay is a serious uncertainty factor. Thus, the number of messages should be reduced and collisions between messages must be avoided to increase the accuracy and precision of time synchronization.

## 3. Enhanced Precision Time Synchronization

### 3.1. Precision Time Synchronization

**Figure 2.**
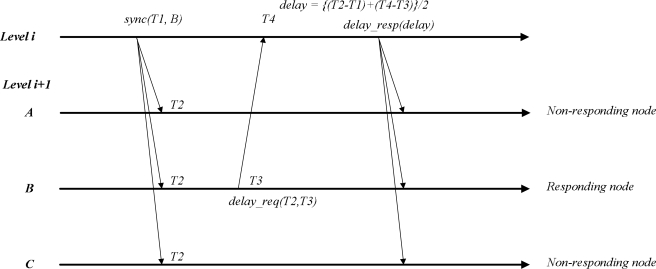
Lightweight time synchronization.

Tsync [12] and LESSAR [13] are lightweight time synchronization protocols. In this section, we briefly introduce light time synchronization protocols. Figure 2 illustrates the concept of these synchronization protocols.

The method uses three message types: *sync, delay_req* and *delay_resp*. Initially, a *sync* message is sent by the sink node, which is level 0, and acts as a root node providing the reference clock. The sink inserts time *T1* into the *sync* message, and each sensor node receives the packet at time *T2* and records their local clock. Then, the sensor node determines the clock offset as *δ = T2* − *T1*. To calculate the delay between the sink and sensor nodes, delay calculations from all child nodes lead to a variety of traffic, which results in inaccurate synchronization. Thus, we assume that the uncertainties in the propagation speeds are the same in different nodes and the uncertainty by the propagation delay is less than other uncertainties such as the send, access, receive time, etc. Under this assumption, in the proposed method, only one child node responds to calculate the propagation delay from the sink node or parent node. The sink determines which node responds to the sink node to measure the delay by consulting its neighbor list. This selection is based on a min-ID selection. Information for the responding node is inserted into the *sync* message. The sensor node receiving the *sync* message first checks whether it itself is target for the message. If so, the sensor node sends the *delay_req* message, including times *T2* and *T3*. Otherwise, it will be discarded. Then, the sink receives the *delay_req* message at time *T4* and records the arrival time of the message. Next, the sink determines the propagation delay between its one-hop children sensor nodes and itself, as demonstrated in Equation (1), and broadcasts it to its one-hop nodes. Finally, the child nodes can correct the propagation delay by receiving the *delay_resp* message from the sink node.

### 3.2. Proposed Precision Time Synchronization

**Figure 3.**
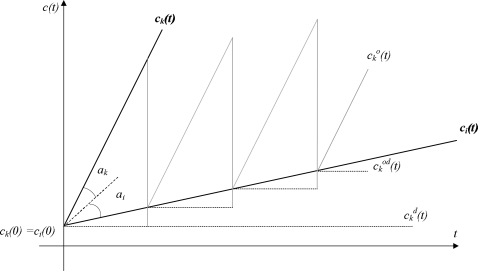
Clock difference by local clock drift.

Although two clocks are initially set to the same frequency via the correction of offset and delay, a difference between them accumulates as time advances [17]. Assume that the local clocks of two nodes, *i* and *k*, are *c_i_(t)* and *c_k_(t)*. If *c_i_(t)* = *c_k_(t)*, the two clocks are synchronized at time *t*. If the algorithm for time synchronization could know the relative offset between *c_i_(t)* and *c_k_(t)* at time *t*, *c_k_(t)* can be synchronized to *c_i_(t)* at each epoch by correcting the relative offset. Figure 3 represents the synchronized clock $c_{k}^{o}(t)$. Although *c_k_(t)* is exactly synchronized to *c_i_(t)* via periodic correction, clock $c_{k}^{o}(t)$ pursues the line derived from a variation of clock *c_k_(t)*, because this synchronization did not consider clock drift. Thus, LESSAR assumed that the clock drift quickly changes. Therefore, they frequently conduct the synchronization procedure, but this eventually decreases the synchronization accuracy because it keeps the channel busy with excessive synchronization messages.

**Figure 4.**
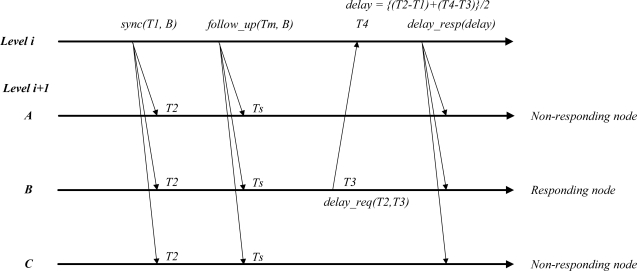
Synchronization protocol for drift correction.

However, the clock drift on a real sensor node does not change quickly as time advances. We verify this condition on an implemented sensor node in the section on the performance evaluation. In this paper, we propose an additional message, *follow_up*, to minimize the clock drift difference between two clocks. As shown in Figure 4, the *follow_up* message is a subsequent message that follows the *sync* message and includes the event-timestamp marking the transmission by the sink node. The subsequent *follow_up* message helps calculate the drift from the sink node. Equation (2) presents the drift compensation correction at the sensor nodes:

(2) $$\begin{array}{cc} \begin{array}{c} \Delta_{m}=T_{m}-T_{1}\\ \Delta_{s}=T_{s}-T_{2} \end{array}, & \Delta_{\mathrm{diff}}=\frac{\Delta_{s}-\Delta_{m}}{\Delta_{m}} \end{array}$$

where Δ*_m_* is the clock drift of the sink node that applies to clocks between *Tm*, which is the time stamp value in a *follow_up* message, and *T1*, which is the time stamp value in the *sync* message. Δ*_s_*, the clock drift of the sensor node, applies to clocks between the arrival time of the *sync* message, *Ts*, and the arrival time of the *follow_up* message, *T2.* Δ*_diff_* indicates the difference between two nodes to be corrected. The proposed approach makes it possible to calculate the drift rate by using only one synchronization procedure, which dramatically reduces the number of messages needed for synchronization. The proposed method should be achieved under assumption that precision time stamping is done.

**Figure 5.**
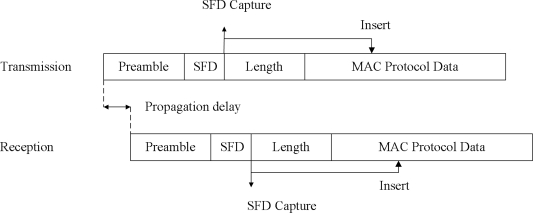
Time stamping point during synchronization procedure.

As previously mentioned, the time stamping point is critical because it affects the time synchronization accuracy. The time stamping point can be any point in the network layers. However, time stamping at an upper layer such as the application layer has the disadvantage that the protocol stack can cause delays that may not be deterministic [22]. In this study, time stamps are taken at the physical layer using a time stamping unit. Figure 5 shows the method for determining the time of an event such as message transmission and reception.

In Figure 5, the time stamping unit detects and time stamps the start of the frame delimiter (SFD) signal from the radio transceiver to minimize the uncertainties occurring at the network protocol stack. We have designed and implemented special purpose hardware. Later, we will describe the precision time protocol used in the hardware that we developed. The proposed synchronization method is a good mechanism in a single hop network. However, a novel approach is needed for the multi-hop pattern in a wireless sensor network. Thus, we also proposed a way reduce the number of messages in a multi-hop network.

### 3.3. Proposed Enhanced Precision Time Synchronization

Tsync and LESSAR seem to minimize the number of messages needed for synchronization in a single hop network. However, they do not include a reduction method for the number of messages in a multi-hop network. Message delivery, including beacon, time sync messages, *etc.*, in wireless networks is usually based on flooding. Flooding-based message delivery can lead to unnecessary, duplicated messages in the network. This section proposes a method to eliminate such duplicated messages and reduce the messages needed for synchronization. The proposed method can also reduce the uncertainty by channel contention because the number of messages needed for packet delivery is fixed in a broadcast domain. The proposed method initially creates a network topology from the sink node for multi-hop time synchronization. The sink assigned as a *root* initiates the topology construction procedure by broadcasting a level discovery packet, which includes a hierarchical level and a route to the sink node. Every sensor node that receives the level discovery packet from the sink is assigned a hierarchical level and a path to the sink in this hierarchical topology, and it records the sensor node-sent level discovery packet to its parent node. Information on the level and route should be updated when the packet undergoes the sensor node. Thus, each node has only one path from the sink. In this case, we ensure that the sensor node belonging to level *i + 1* can communicate with a device belonging to its parent, which has level *i*.

**Figure 6.**
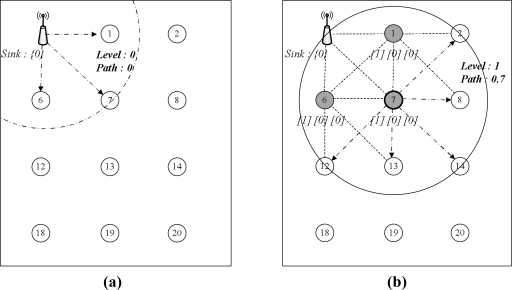

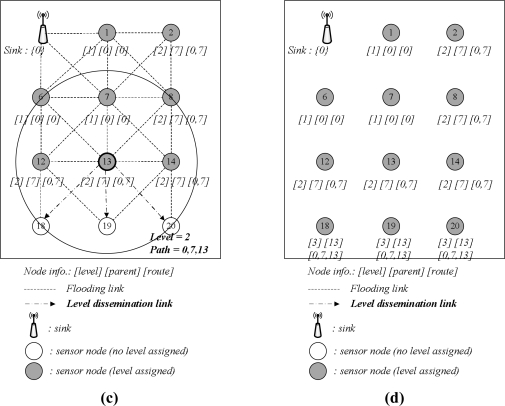
Topology construction procedure for multi-hop time synchronization. **(a)** Level 0 dissemination; **(b)** level 1 dissemination; **(c)** Level 2 dissemination; **(d)** completed topology.

**Figure 7.**
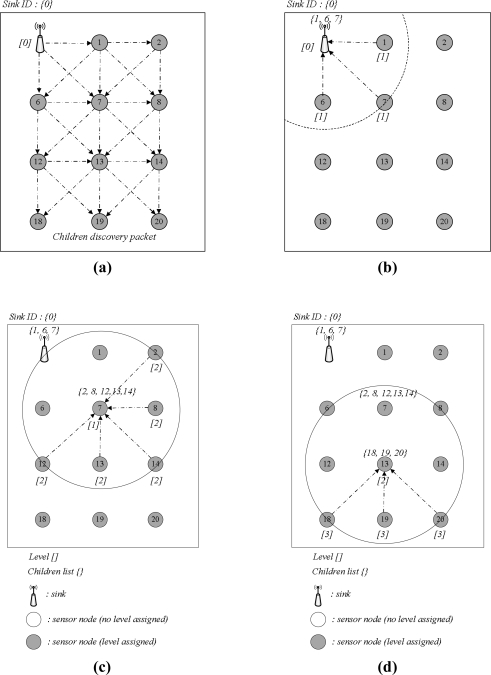
Children discovery phase.

Figure 6 illustrates an example of the topology construction procedure. The sink node sends level and route information, and the sensor node receives message updates and maintains its level, route and parent information. For example, node IDs 1, 6, and 7 indicate level 1, route to sink *[0]*, where 0 is the sink ID, and parent ID 0, respectively. The sink node sends a child discovery packet to let every node discover its child nodes in the hierarchical topology, once the network topology is constructed. Figure 7 illustrates the procedure for the child discovery phase. In Figure 7(a), the sink broadcasts the child discovery packet to the network. Any sensor node receiving the packet responds to the sink or its parent node, as shown in Figure 7(b), and the sink or the parent node receives the response message and records the child sender to its child list.

Next, child nodes with level 1 rebroadcast the child discovery packet and then collect their child nodes, as shown in Figure 7(c). This process is continued until every node is discovered. This child list is utilized in selecting candidate nodes, which are the responding nodes used to send the *delay_req* message for calculating the propagation delay between levels *i* and *i + 1.* Time synchronization in the hierarchical topology is achieved by flooding; the synchronization procedure is the same as with a single-hop synchronization pattern. However, flooding-based synchronization in a multi-hop and distributed network produces traffic in proportion to the number of sensor nodes. For instance, in Figure 7, a single message can be delivered via only three forwardings, while flooding is performed nine times. Excessive traffic from flooding causes duplicated messages, channel contention and message collision.

**Figure 8.**
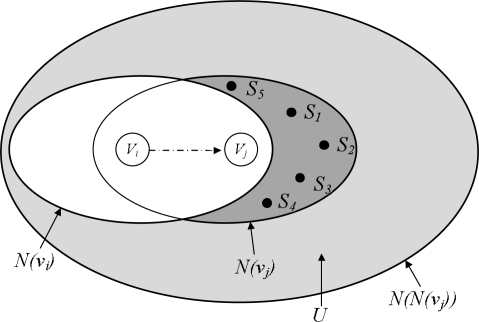
Selective forwarding.

We propose a packet delivery process based on the dominant-pruning algorithm [23] to avoid excessive messages during topology construction, children discovery, and time synchronization. This algorithm determines from the prior sender whether the next node forwards the packet. Assume that node *V_j_* receives the packet from node *V_i_*, and *V_j_* is included in the forward list of the packet. *N(V_i_)* is the set of one-hop neighbors from *V_i_*, and *N(V_j_)* is the set of one-hop neighbors from *V_j_*. Sensor node *V_j_* makes a forward list to cover neighbors within two hops. The set of two-hop neighbors is represented as *N(N(V_j_)).* When node *V_i_* sends the packet to node *V_j_*, *V_i_* inserts a set of its one-hop neighbors, *N(V_i_)*, into the packet. Then, node *V_j_* determines a forward list. The selection of the next node for packet delivery is shown in Algorithm 1 and Figure 8.

**Algorithm 1.** Selective forwarding.

Let *F* = *Ø, Z = Ø, K* = {*S_1_*, *S_2_*, … ,*S_n_*}
where *S_k_* = *N(V_k_)* ∩ *U*
if *V_j_* == *sink node*
*U = N(N(Vj))* − *N(Vj)* − *Vj*
else
*U = N(N(V_j_))* − *N(V_j_)* − *N(V_j_)*
end if
while (*Z != U*)
Find the set *S_k_* whose size is the maximum within set *K*
*F* = *F* ∩ {*V_k_*}, *Z* = *Z* ∩ {*S_k_*}
*K* = *K* − {*S_k_*}, *S_l_* = *S_l_* − *S_k_* for all *S_l_* ∈ *K*
end while

## 4. Performance Evaluation

This section evaluates the enhanced precision time synchronization performance for WSNs. The performance evaluation is separated into two phases: a simulation and experiment.

### 4.1. Simulation

The performance evaluation criteria can be classified into accuracy, precision, and the number of messages generated for the synchronization procedure. Accuracy denotes the offset from the reference clock and can be determined as the average offsets by sensor nodes in the network. Precision is an error range and can be derived from a standard deviation or a root mean square (RMS). Finally, the number of messages is measured by accumulating the message frequency occurring in the overall network. we use the TrueTime simulator [24] based on Matlab/Simulink to compare performances. Table 1 shows the simulation setup. The network size is 500 × 500 m, CSMA is used for MAC and the initial offset and clock drift are set to 2 s and 20 PPM, respectively.

**Figure 9.**
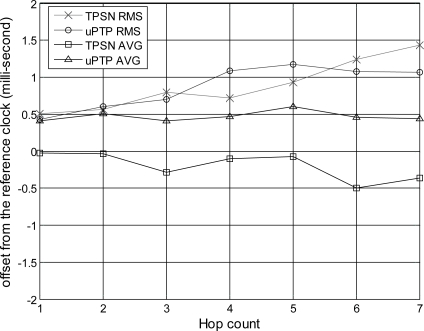
Accuracy and precision *vs*. hop distance.

First, we evaluate the accuracy and precision of the time synchronization based on the hop distances from the sink node, the reference clock. Figure 9 illustrates the accuracy and precision according to hop distance from a single hop to a 7-hop distance.

TPSN was used to evaluate the proposed method. We denote the proposed method as “*uPTP*” to easily understand the results. This graph implies that the closer the offset is to zero, the more precise and accurate the synchronization is; a zero value indicates perfect synchronization with the sink node. A positive value implies that the sensor node runs faster than the sink node used to provide the reference clock, whereas a negative value implies that the sensor node runs slower than the sink node. As mentioned earlier, the root mean square (RMS) denotes the precision and the average (AVG) is the accuracy of the synchronization between the sink and sensor nodes. A comparison of the proposed method with TPSN, which is known as the standard for WSNs, does not reveal a large difference in terms of precision, and with both protocols experiencing an increase in the precision error when the hop distance increases. However, both methods diverge from zero in terms of accuracy. The average accuracy of the proposed method depicts negative values, while that of TPSN shows positive values. This phenomenon occurs because of the asymmetric communication link when calculating the propagation delay between levels *i* and *i + 1*. That is, TPSN is initiated by level *i + 1*, while the proposed method is initiated by level *i*. This results in a difference between the two protocols, as shown in the graph. The graph summarizes that when there is a one hop distance in the TPSN, the RMS is approximately 507 μs, and the average offset is −24 μs. When there is a 7-hop distance, TPSN shows an RMS of 1,431 μs and an average of −364 μs. The proposed method has an RMS of approximately 431 μs and an average error of 411 μs in a single hop distance. The proposed method shows an RMS of 1,066 μs and an average of 444 μs when increasing up to a 7-hop distance. Thus, this graph shows that the difference between the two protocols in a sparse environment is subtle.

Second, we evaluate and analyze the performance according to network scalability. Table 2 summarizes the system setup for the evaluation to measure the performance based on network density. The network size is 500 × 500 m, the number of nodes ranges from 40 to 70, the communication range is set to 120 m and the nodes are deployed uniformly in a random pattern. A network partition occurs when the number of nodes is below 30 because of the communication range limitation.

**Figure 10.**
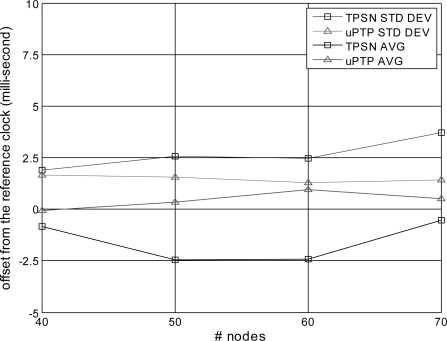
Accuracy and precision *vs.* number of nodes.

Figure 10 illustrates the accuracy and precision based on network density. When there are 40 nodes, TPSN has an average offset of −0.83 ms and a standard deviation of 1.88 ms, while the proposed method has an average offset of −0.065 ms and a standard deviation of 0.35 ms. When the number of nodes increases to 70, the proposed method has an offset of 1.42 ms, while that of TPSN increases up to 3.7 ms. This result was caused by TPSN having numerous random back-off uncertainties from excessive messages, while the proposed method reduced the random back-off uncertainties by using fewer messages for synchronization. That is, the number of messages in TPSN increases based on the number of nodes, which increase the number of retransmissions, whereas the proposed method experiences only a small increase in the number of messages, which reduces the uncertainty by retransmission.

**Figure 11.**
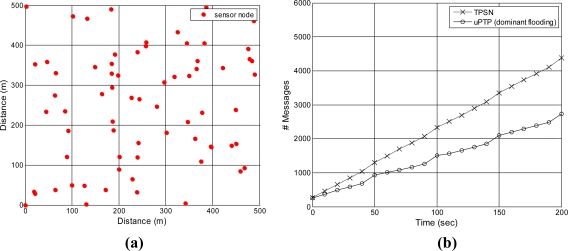
Number of messages *vs.* time advancement. **(a)** sensor topology; **(b)** number of messages.

Finally, this study measured the network traffic for time synchronization as time advanced. We used a 70 node topology, topology construction was conducted every 50 s, and the time synchronization procedure was performed every 10 s to measure traffic. Figure 11 shows that the proposed method generates 1,504 messages, while TPSN generates 2,326 messages after 100 s. The difference between the two methods increases as time advances. After 200 s, TPSN has 4,380 messages, while the proposed method results in 2,733 messages. We can easily expect the precision to increase and the traffic to decrease in dense environments using the proposed approach.

### 4.2. System Implementation

**Figure 12.**
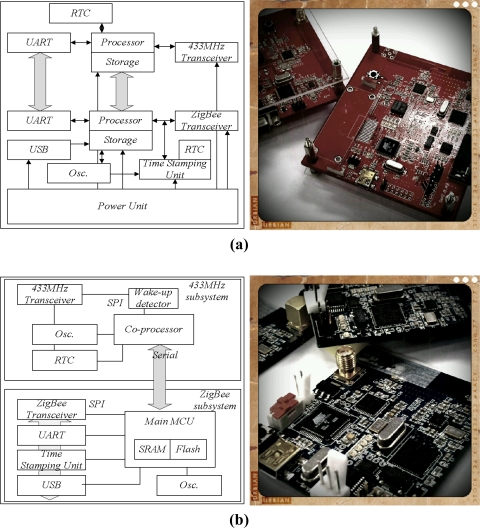
Sink node and sensor node. **(a)** sink node; **(b)** sensor node.

We evaluated the proposed time synchronization protocol via a simulation. However, because this simulation did not provide exact information about the time stamping unit, clock drift, *etc.*, we implemented the sink and sensor nodes using Commercial-Off-The-Shelf (COTS) technology, as shown in Figure 12. Figure 12(a,b) depict the diagrams and prototypes for the sink and sensor nodes, respectively. The sink and sensor nodes are each separated into two parts: a 433-MHz subsystem on AVR and a ZigBee subsystem on ARM. The ZigBee subsystem used for communication control, including time synchronization, and the 433-MHz subsystem is in charge of data exchange and aggregation. Communication between the two subsystems is achieved via a serial interface. The 433-MHz subsystem uses the ATmega128L processor and TI’s CC1100 RF transceiver. This 433-MHz subsystem is able to operate at a maximum frequency of 8 MHz, providing reasonable processing power to explore a wide variety of applications. The Atmega128L processor provides sufficient memory resources for a wide range of experiments. The on-chip memory includes 4 KB of RAM, 4 KB of EEPROM and 128 KB of flash memory. General purpose I/O pins and serial ports such as RS-232 and SPI are provided by the processor.

**Figure 13.**
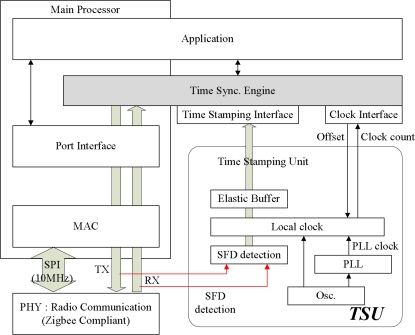
Precision time stamping unit.

The ZigBee subsystem uses an ARM-based microcontroller (AT91SAM7S) and a CC2420 RF transceiver. The ZigBee subsystem operates at a maximum frequency of 55 MHz. The processor is based on a 32-bit RISC architecture, and it provides 256 KB of flash memory, 64 KB of SRAM, and various peripherals such as UART, USB 2.0, and SPI. The hardware prototype consists of a processing unit, communication unit and time processing unit. The time synchronization protocol is implemented in the processing unit and used for synchronization of the sink node and sensor nodes. The time stamping unit time stamps in the physical layer and provides the system clock, which synchronizes the sink node and sensor nodes. The time stamps read by the time stamping unit are delivered to the processing unit. Figure 13 shows the architecture and prototype of the time stamping unit.

### 4.3. Experimental Result

**Figure 14.**
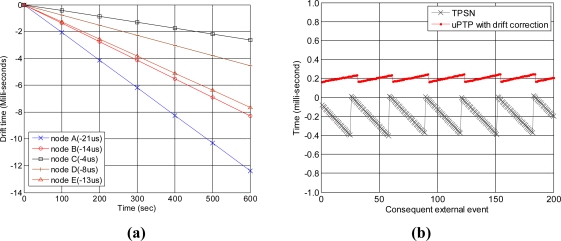
Analysis of clock drift of sensor nodes from sink node. **(a)** clock drift rate of sensor nodes; **(b)** clock offset *vs.* time advance.

We compared and analyzed the clock drift rate of the sensor nodes from the sink before performing an evaluation of the sensor nodes. A general crystal oscillator with a clock drift of 20 parts per million (PPM) was chosen as the clock source of the sensor nodes. The results of an analysis are shown in Figure 14(a). The graph shows that five sensor nodes drift from the sink node and have different drift rates than the sink node. Sensor nodes A, B, C, D and E drift by approximately −21 μs, −14 μs, −4 μs, −8 μs, and −13 μs per second, respectively. The negative values indicate that the sensor nodes run slower than the sink node, while a positive value means that a sensor node runs faster than the sink node. As shown in the graph, the clock difference increases as time advances. Thus, clock drift correction is needed for accurate and precise time synchronization. Figure 14(b) presents the clock offset of the proposed method and that of TPSN.

**Figure 15.**
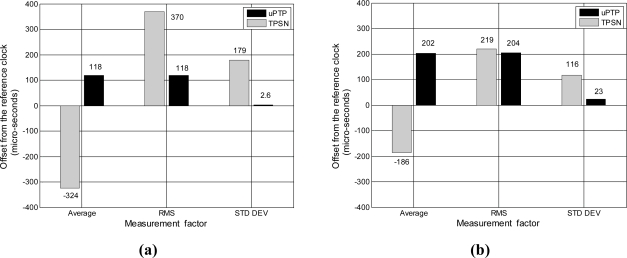
Synchronization results by clock drift correction. **(a)** responding node; **(b)** non-responding node.

We performed experiments on the developed sensor node to implement the physical phenomenon. Table 3 and Figure 15 show the results for the accuracy and precision using the precision time stamp and clock drift correction. The experimental condition was that the synchronization interval was every 30 s in a single hop network. The performance evaluation is separated into two parts, a responding node and non-responding node, because the proposed method exchanges the time stamp with only one child node to calculate the propagation in the hierarchical tree. The results show that in TPSN, which has precision time stamping but does not correct clock drift, the responding node has an average offset of −324 μs and a standard deviation of 179 μs, while the non-responding node has an average offset of −186 μs and a standard deviation of 116 μs. However, in the proposed method, which has precision time stamping and corrects clock drift, the responding node has an average offset of 118 μs and a standard deviation of 2.6 μs, and the non-responding node has an average offset of 202 μs and a standard deviation of 23 μs. High precision time synchronization via clock drift correction is achieved under the assumption that the node can time stamp the clock and arrival time of the message without delay and jitter.

## 5. Conclusions

In this paper, we have proposed an enhanced precision time synchronization for wireless sensor networks. Uncertainty, which has a harmful influence on the accuracy of time synchronization, can occur at all layers in a network protocol stack. To provide precision and accurate time synchronization, we analyzed the delay and jitter caused at a network protocol stack and minimized delay and jitter. First, we proposed a method to scatter time information across the WSN field, minimize jitter by random back-off and provide precision time synchronization by dramatically reducing network traffic. Second, we showed how sensor nodes could be sourced from a local crystal oscillator that drifts as time advances. Thus, the time difference will be increased over time. In the proposed method, sensor nodes linearly pursue the line of the reference clock by correcting their clock drift. This study also designed and implemented a precision time stamping unit and evaluated its performance. In addition, to demonstrate the superiority of the proposed time synchronization method for WSNs, this study implemented a special sink node and a sensor node. According to the results of the performance evaluation, the proposed method greatly reduced the number of messages used for synchronization and had 5-fold better performance than a traditional time synchronization protocol in terms of accuracy.

The proposed method can be used as a foundation for many applications requiring strict clock synchronization. In addition, it can be extended to other radio frequencies, as well as IEEE 802.15.4, for accurate clock synchronization. This represents a milestone for precision time synchronization for a wide-scale wireless sensor network. We expect the proposed method to have a significant impact on the efficiency of many sensor applications, including those used for field surveillance, environment or habitat monitoring, localization, and asset management. Future work will include applications of the introduced system to real world scenario.

## Figures and Tables

**Table 1.** Experimental setup for evaluation.

**Field area**	**500 × 500 m**
MAC	802.15.4 (CSMA)
Data rate	250 kbps
Sync interval	10 s
Packet size	28
Initial offset	2 s
Clock drift	20 PPM

**Table 2.** Experimental setup for evaluation with number of nodes.

**Field area**	**500 × 500 m**
Deployment	Uniformly random
# Nodes	40, 50, 60, 70
Communication range	120 m

**Table 3.** Synchronization results via clock drift correction.

	**Responding node**			**Non-responding node**		
	
	AVG	RMS	Std Dev	AVG	RMS	Std Dev
TPSN	−324	370	179	−186	219	116
uPTP	118	118	2.6	202	204	23

Unit: microseconds (μs).
